# The Function of the Metals in Regulating Epigenetics During Parkinson’s Disease

**DOI:** 10.3389/fgene.2020.616083

**Published:** 2021-02-02

**Authors:** Xiangzhen Wei, Menghua Cai, Lifang Jin

**Affiliations:** ^1^Department of Biological Sciences, Xi’an Jiaotong-Liverpool University, Suzhou, China; ^2^Department of Immunology, Institute of Basic Medical Sciences, Chinese Academy of Medical Sciences and School of Basic Medicine, Peking Union Medical College, Beijing, China; ^3^Department of Biological Sciences, Shaoxing University, Shaoxing, China

**Keywords:** Parkinson’s disease, epigenetic, metal, DNA methylation, synucleinopathy

## Abstract

Parkinson’s means Parkinson’s disease, a chronic degenerative disease of central nervous system. The main area which is affected by this disease is motor system. Since it firstly founded by James Parkinson in his 1817 publication, nowadays, people still have lots of questions about this disease. This review mainly summarizes the epigenetics of Parkinson’s. DNA methylation is one of the epigenetic mechanisms of Parkinson’s. During the development of disease, global hypomethylation, and hypermethylation happen in different areas of patients. Another epigenetic mechanism is histone modification. People believe that some metals can induce Parkinson’s disease by modulating epigenetic mechanisms. This review summarizes the relationships between different metals and Parkinson’s disease. However, the specific roles of most metals in epigenetics are still unknown, which need further research.

## Introduction

Parkinson’s disease (PD) is a common neurodegenerative disease (ND) with movement disorder (de Lau and Breteler, 2006), which is characterized by the progressive and substantial loss of dopaminergic neurons in substantia nigra. The hallmark of PD is the accumulation of cytoplasmic proteins, especially α-synuclein, a major competent of Lewy Bodies (LB). Moreover, PD patients show symptoms of resting tremor, slow movement, muscular rigidity, and postural instability (Nataraj and Rajput, 2005). Point mutations in α-synuclein are known to cause familial PD, accounting for about 5% of PD patients (Chang and Fox, 2016). Idiopathic PD, which accounts for about 90–95%, usually refers to a syndrome characterized by late-onset Parkinsonism (Ceravolo et al., 2009). In this review, we discuss the roles of α-synuclein in PD, especially its synthetic effect. Then we discuss the epigenetics of Parkinson’s, mainly about DNA methylation. Histone modification also plays an important role in PD. However, the direct evidence between histone modification and metals is lacking. At last, we summarize the roles of different metals in Parkinson’s.

### Synucleinopathy

Intriguingly, α-synuclein was first found in the brains of AD patients (Uéda et al., 1993), and then was viewed as the main part of Lewy bodies in PD (Irizarry et al., 1998). Deas et al. (2016) reported that oligomeric α-synuclein suppressed the production of glutathione and showed the toxicity of neurons, while fibrillar forms could not. In a mice model expressing human α-synuclein, a progressive memory loss and motive dysfunction was observed (Fernagut and Chesselet, 2004).

There are several explanations for α-synuclein neurotoxicity. All of them lead to the activation of the apoptosis pathway and neuron death ultimately (Scarlet et al., 2015). In PC12 cell lines, the overexpression of the A53T mutant α-synuclein resulted in the death of 40% of neurons through the following mechanisms:

(1) Inducing the releasing of mitochondrial cytochrome C, leading to the breakdown of the respiratory chain, leakage of ROS, and mitochondrial dysfunction. In human neuroblastoma cells, overexpression of α-synuclein resulted in elevated ROS and inhibition of the respiratory chain (Parihar et al., 2009).
(2) Increasing endoplasmic reticulum (ER) stress (Smith et al., 2005). In SHSY cells, overexpression of α-synuclein also stimulated the release of mitochondrial cytochrome C (Parihar et al., 2008). In a mouse model of α-synuclein overexpression, endoplasmic reticulum stress, a mechanism triggered by misfolding proteins, showed a synthetic effect with α-synuclein in the onset of PD (Colla et al., 2012a, b).
(3) α-synuclein can form a pore in membranes, leading to the disturbance of calcium metabolism and cell death. Through single-channel electrophysiology, pores formed by α-synuclein were observed directly in the lipid membranes of cells (Schmidt et al., 2012).
(4) In a dopaminergic-like cell line, α-synuclein induced the formation of leak channels reminiscent (Feng et al., 2010). In the cell line model, α-synuclein facilitated the formation of a pore in the membrane, disturbing ion homeostasis, and leading to neuron death (Danzer et al., 2007).
(5) Furthermore, α-synuclein disturbed neurotransmitter release by inhibiting the trafficking and recycling of synaptic vesicles (Wang et al., 2014).

## Epigenetics

### DNA Methylation

DNA methylation is the earliest characterized chromatin modifications. Since the majority of methylation occurs in CpG motifs, which are enriched in promoters. The methylation of CpG dinucleotides at the 5′ position on the pyrimidine ring, to form 5-methylcytosine (5-mC), can disrupt the cell’s transcriptional machinery by blocking the binding of transcription factors and attracting methyl-binding proteins that initiate chromatin compaction and bring about gene silencing (Lunnon and Mill, 2013). So methylation results in gene silencing while de-methylation causes gene activation.

Lumine et al. (2010) reported global hypomethylation in substantia nigra in PD patients. Moreover, intron 1 of α*-synuclein* showed hypo-methylation, leading to the over-expression of α-synuclein (Jowaed et al., 2010). On the other hand, L-dopa stimulated the hypermethylation of intron 1 of α-synuclein to suppress its expression. This might be a new explanation for the positive effects of L-dopa in PD patients (Schmitt et al., 2015). PD patients demonstrated a lower level of DNA methylation in *SNCA* and *PARK2* genes compared with controls (Eryilmaz et al., 2017).

## Metals

Previous researches found that there are relationships between metals and PD. On the one hand, metals, especially heavy metals are usually regarded as neurotoxins, because they can cause neuronal death by oxidative stress. For example, both iron and copper can induce oxidative stress and cause damage to neurocyte. People have a relatively clear understanding of the pathophysiology of different metals. On the other hand, recently scientists found metals can regulate epigenetics during PD. Understanding the roles of metals in epigenetics of PD might help people find a cure for PD. However, there is a lack of relevant research. The following summarizes the relevant contents about metals (Table 1).

**TABLE 1:** Epigenetic and toxic effects of different metals in Parkinson’s.

	Epigenetic	Principal target of metal-induced toxicity	Pathophysiology
Lead (Pb)	Cause LINE1 promoter hypermethylation	LINE1/nervous system	Oxidative stress, mitochondria dysfunction, Ca^2+^ homeostasis disruption (Chen et al., 2016a)
Mercury (Hg)	Tau phosphorylation	Tau protein/mitochondria	Loss of dopamine receptors, tubulin degeneration, axon degeneration and glutathione depletion, higher amyloid-β level (which promotes α-synuclein aggregation) (Bjorklund et al., 2018)
Copper (Cu)	Oxidative stress mechanisms, alpha-synuclein oligomerization and Lewy body formation, as well as GABA-A and NMDA receptor neurotransmission modulation	Cytochrome/mitochondria/brain	Increased generation of ROS, DNA, and mitochondrial dysfunction
Manganese (Mn)		Globus pallidus in the basal ganglia	Impairment of dopaminergic, glutamatergic, and GABAergic transmission, as well as mitochondrial dysfunction, oxidative stress and marked neuroinflammation (Cicero et al., 2017)
Aluminum (Al)	Al facilitates the formation of alpha-synuclein fibril by activating monoamine oxidase B	Monoamine oxidase B	Calcineurin β protects brain after injury by activating the unfolded protein response. Neurobiology of disease (Chen et al., 2016b)
Iron (Fe)	Up-regulation of divalent metal transporter 1 (DMT1) (Zhang et al., 2009)	Glutamate receptors (Lau and Tymianski, 2010), Cu protein, Ceruloplasmin (Cp) (Jiang et al., 2015)	Iron stimulates the formation of intracellular aggregates of α-synuclein and promotes oxidative damage.
Zinc (Zn)	Accumulation of alpha-synuclein	Autophagy-lysosomal pathway (Cicero et al., 2017)	Tsunemi and Krainc (2014) reported that the loss of PARK9 leads to the dyshomeostasis intracellular zinc levels, which contributes to lysosomal dysfunction then leading to the accumulation of alpha-synuclein.

### Lead (Plumbum, Pd)

Neurological damage caused by lead was found in 2006 by Monnet-Tschudi et al., they found that Lead exposure causes severe swelling and loss of neurons in the central nervous system and peripheral nervous system (Monnet-Tschudi et al., 2006).

In a case-control study of 121 PD patients and 414 controls, there was a dose-effect relationship between occupational exposure of lead and the risk of PD (Coon et al., 2006). In a case-control study of 330 PD patients (216 men, 114 women) and 308 controls (172 men, 136 women), there was a dose-effect relationship between bone lead and the risk of PD (Weisskopf et al., 2010). Wright et al. (2010) also reported an association between lead exposure and LINE1 hypomethylation. Li et al. (2013) also reported an inverse association between lead exposure and LINE1 promoter hypermethylation in a case-control study.

### Mercury (Hg)

Mercury is also a neurotoxin that can damage neurons (Azevedo et al., 2012). In a case-control study of 54 idiopathic PD patients and 95 controls, there was a dose-effect association between the risk of PD and blood mercury (Ngim and Devathasan, 1989).

Mercury exposure led to DNA methylation changes in whole blood cells (Hanna et al., 2012). In SH-SY5Y cells, mercury also disturbed the clearance of Aβ plaques by suppressing the activity of neprilysin (Miguel et al., 2015). Mercury showed neural toxicity since APOE4 owned a weak combination with mercury (Mutter et al., 2004). Olivieri et al. (2010) reported that mercury stimulated the expression of Aβ and phosphorylation of tau. In PC12 cells, mercury promoted the expression of Aβ and inhibited clearance at the same time (Song and Choi, 2013).

### Copper (Cu)

In a case-control study of 144 patients with idiopathic PD and 464 controls, individuals with more than two decades of copper exposure showed a significantly higher association with risk of PD (*OR* = 2.49, 95% *CI* = 1.06, 5.89) (Gorell et al., 1997). There was a decreased copper concentration in substantia nigra of PD patients (Dexter et al., 1991). Cu and α-synuclein showed the synthetic effects in the inhibition of protein degradation pathways, especially the Ubiquitin Proteasome System (UPS) (Anandhan et al., 2015). To facilitate the accumulation of Aβ through inhibiting its transport, Cu facilitates Aβ accumulation by inhibiting clearance and stimulating production (Singh et al., 2013).

### Manganese (Mn)

Manganese has manganese toxicity through impairing motor function and damaging substantia nigra and other basal ganglia nuclei by amplifying the risk of PD (Aschner and Nass, 2006). The pathology of Manganese induced Parkinson’s disease is different from other idiopathic forms. In a case-control study of 144 patients with idiopathic PD and 464 controls, individuals with more than two decades of manganese exposure showed a significantly higher association with PD (*OR* = 10.61, 95% *CI* = 1.06, 105.83) (Gorell et al., 1997). Wang et al. found a dose-effect relationship between exposure to manganese through inhalation and symptoms from extrapyramidal system dysfunction (Wang et al., 1989). Mn (II) inhibited the functions of mitochondria, which lead to the death of neurons due to energy insufficiency (Gunter et al., 2010). Manganese was associated with DNA methylation. Researchers have found a new method to identify the risk resulting from toxic metal exposure by measuring the level of DNA methylation. There is an important relationship between DNA methylation aging biomarkers and the concentration of some metals. For example, if the concentration of Mn in urine increase by 1 ng/mL, PhenoAge will increase by 9.93 years (Nwanaji-Enwerem et al., 2020).

### Aluminum (Al)

In a case-control study of 200 PD patients and 200 controls, there were significantly higher levels of aluminum in the substantia nigra of PD patients than controls (Altschuler, 1999). Results from Uversky offered one explanation for the cause-effect association between Al and PD. Al activated monoamine oxidase B, an enzyme that facilitated the formation of alpha-synuclein fibril in PD (Uversky et al., 2001).

### Iron (Fe)

In a case-control study of 892 participants, the concentration of iron in toenails showed a positive association with the level of LINE-1 methylation (Tajuddin et al., 2013). A test of total iron concentration in the substantia nigra of 17 parkinsonian and 29 control samples showed that the substantia nigra of PD patients contained a higher level of iron (Wypijewska et al., 2010). There was an elevated total iron level and decreased ferritin content in the substantia nigra of PD patients (Dexter et al., 1991). Iron deficiency inhibited the translation of a-synuclein mRNA (Febbraro et al., 2012). Murine treated with a high concentration of iron showed symptoms of PD when aging (Kaur et al., 2007). However, another study that explored the relationship between iron in diet and risk of PD indicates that dietary iron intake does not increase the risk of PD (Cheng et al., 2015). The different results of the two experiments may result from the amount of iron and different research models. Furthermore, iron has other functions that may also induce PD. Firstly, iron has alpha-synuclein toxicity. Secondly, iron can induce a Fenton-Haber-Weiss reaction, finally causing oxidative stress (Hellman and Gitlin, 2002; Caudle et al., 2012). Both of these two functions can cause damage to brain cells and damage mitochondria, but the specific link between them and Parkinson’s is unknown, and there is still a need for further research.

### Zinc (Zn)

In a case-control study of 423 PD patients and 205 controls, patients showed significantly more exposure to zinc (95% CI, 1.51–90.90) (Pals et al., 2003). Zinc induced a significant decrease of Aβ solubility and an increase of its ability to resist tryptic cleavage at the secretase site (Bush et al., 1994). Zinc facilitated neuro-filament phosphorylation in the absence of the p70 S6 kinase in N2a cells (Bjorkdahl et al., 2005). There was an elevated zinc concentration in the substantia nigra of PD patients (Dexter et al., 1991).

### Cerium (Ce)

Cerium is an interesting metal that has been the subject of great research interest in recent years. Cerium was proved to have a negative effect on DNA methylation, in other words, Cerium is likely to induce PD. However, another compound of Cerium, cerium oxide nanoparticles (CeO2 NPs) shown positive effects and could cure some neurodegenerative diseases including PD. In a study researching the relationship between concentrations of Cerium and DNA methylation in blood, samples were collected from people who lived around an e-waste disassembling factory in China. In this study, there was a negative correlation between the Ce concentration of pre-workers and global DNA methylation (5-mc) in Pearson correlation and multiple linear regression analysis, *r* = −0.51, *p* = 0.01. Therefore, the concentration of Ce in blood was significantly negatively correlated with global DNA methylation. DNA hypomethylation usually relates to chromosome instability and increased mutation events by affecting the intergenomic and intron regions of DNA, especially repeat sequences and transposable elements. This suggests that Ce plays a key role in DNA methylation reduction. Taking into account that many researchers have also shown that PD is regulated by DNA methylation, it can be concluded that Cerium may increase the risk of PD by DNA methylation reduction (Li et al., 2020). However, the limitation of this research is the lack of experimental models to validate findings in Chinese workers. In other research, scientists found that CeO2 NPs can reduce α-synuclein induced toxicity in a yeast model based on the heterologous expression of the human α-synuclein. To be specific, CeO2 NPs can suppress α-syn-induced mitochondrial dysfunction, reduce the production of reactive oxygen species (ROS) in yeast cells and absorb α-synuclein directly on its surface (Ruotolo et al., 2020).

## Conclusion

Several intriguing points should be mentioned about the roles of metals in PD development because they play both pathological and protective effects. According to previous research, some metals play both negative and positive roles in PD development, such as cerium and copper. To be specific, excessive Cu can induce the generation of ROS, causing DNA and mitochondrial dysfunction. However, Cu plays a protective role in PD patients who are Cu-deficient. Cerium was also proven to have a negative effect on DNA methylation while cerium oxide nanoparticles are used to cure PD.

Another interesting point relates to the different valence states of metals that have different toxicities. For example, divalent Fe is important in PD development because it can induce neuronal death by oxidative stress. But it can become non-toxic if divalent Fe is oxidized to trivalent Fe by ceruloplasmin and hephestin. Researchers have validated this in a double knockout mouse lacking both cap and hephestin. Considering that many metals induce Parkinson’s disease by oxidative stress to cause neuronal death, we believe further research could focus on the translation of metal valence, and this might be a new method of curing PD.

## Author Contributions

XW drafted manuscript. MC edited and revised manuscript. LJ approved final version of manuscript. All authors contributed to the article and approved the submitted version.

## Conflict of Interest

The authors declare that the research was conducted in the absence of any commercial or financial relationships that could be construed as a potential conflict of interest.
